# Teaching Elderly Adults to Use the Internet to Access Health Care Information: Before-After Study

**DOI:** 10.2196/jmir.7.2.e19

**Published:** 2005-06-30

**Authors:** Robert J Campbell, David A Nolfi

**Affiliations:** ^2^The Gumberg LibraryDuquesne UniversityPittsburghPAUSA; ^1^Rangos School of Health SciencesDepartment of Health Management SystemsDuquesne UniversityPittsburghPAUSA

**Keywords:** Aged, health, patient participation, health information system, Web-based services, Internet, attitude to health, physician-patient relations

## Abstract

**Background:**

Much has been written about the Internet's potential to revolutionize health care delivery. As younger populations increasingly utilize Internet-based health care information, it will be essential to ensure that the elderly become adept at using this medium for health care purposes, especially those from minority, low income, and limited educational backgrounds.

**Objective:**

This paper presents the results of a program designed to teach elderly adults to use the Internet to access health care information. The objective was to examine whether the training led to changes in participant's perceptions of their health, perceptions of their interactions with health care providers, health information–seeking behaviors, and self-care activities.

**Methods:**

Participants attended a 5-week training course held in public libraries and senior community centers within the greater Pittsburgh and Allegheny County region. Classes within each seminar lasted 2 hours and consisted of lecture and hands-on training. Baseline surveys were administered prior to the course, 5-week follow-up surveys were administered immediately after the course, and final surveys were mailed 1 year later. Instruments included the Multidimensional Health Locus of Control (MHLC) Scale, which measures three domains of locus of control (internal, external, and chance); the Krantz Health Opinion Survey (HOS); and the Lau, Hartman, and Ware Health Value Survey. Two additional questionnaires included multiple choice and qualitative questions designed to measure participants' Internet utilization and levels of health care participation. The Health Participation Survey was administered with the baseline survey. The Internet Use Survey was administered at the 1-year mark and contained several items from the Health Participation Survey, which allowed comparison between baseline and 1-year responses.

**Results:**

Of the 60 elderly adults who began the training course, 42 (mean age 72) completed the entire 5-week training program and the 5-week follow-up questionnaire administered immediately after the program, and 27 completed the 1-year follow-up survey. Statistically significant differences were found between baseline and 5-week follow-up results for MHLC chance subscores in males (*P* = .02) and females (*P* = .05), as well as total HOS information seeking scores (*P* = .05). However, these statistically significant findings disappeared when all 60 original participants were included using a “last observation carried forward” imputation. No statistically significant changes were found between baseline and 5-week follow-up surveys for MHLC external (*P* = .44) and internal (*P* = .97) locus of control scores in both genders, or for the HOS behavioral involvement subscale (*P* = .65).

**Conclusions:**

We failed to show robust before-after effects for most of the outcomes measured. Elderly adults may be willing to use the Internet as a source for general health information; however, when making decisions about their health care, our participants seemed to adhere to a physician-centered model of care. Demographic and situational variables may play a large role in determining which seniors will use the Internet for making behavioral decisions about their health care and in which scenarios they will do so.

## Introduction

The days of the physician-centered, paternalistic model of health care [1], when physicians seemingly provided all answers and all direction, are rapidly fading. Although many health care systems in industrialized countries continue to move toward a shared decision-making model, many seniors learned to interact with their health care providers when the paternalistic model was prevalent. To become independent consumers of health care, seniors must learn to find the health information needed in order to participate in the shared decision-making model. As increasing numbers of seniors go online, the Internet can provide needed health information, but seniors must become both health and health information literate. More research is needed to determine whether Internet use increases seniors' levels of participation, alters their decision-making processes, and most importantly, whether it has a positive impact on seniors' overall health.

### The Digital Divide

Use of the Internet continues to grow exponentially across all age groups in the United States. Fox [2] reports that overall, 77% of 18- to 29-year-olds, 75% of 30- to 49-year-olds, 58% of 50- to 64-year-olds, and 22% of adults 65 and older have access to the Internet. Furthermore, Fox and Fallows [3] report that more than 80% of adult users (or 93 million) have searched the Internet for health information. Of that 93 million, roughly 5 million adults age 65 and older have used the Internet to access and use health care information. Although the discrepancy in Internet use among age groups decreases each year, a large gap exists between seniors who frequently use the Internet to find health care information and those who do not. This gap is of grave concern because the move toward managed care places a greater burden on patients to make decisions about their own health care. Furthermore, US government agencies are now beginning to place an increasing amount of information relevant to Medicare and other programs on the Internet (eg, one option to sign up for the Medicare drug benefit card is to register via the Internet). Seniors who lack access to the Internet as well as the skills necessary to find, retrieve, and evaluate information are at a distinct disadvantage in managing their health care.

Of the 22% of US adults aged 65 and older using the Internet, it is estimated that 66% use the Internet to locate health information [2]. Initial studies suggest the majority of senior users are highly educated white females, with high economic standing, who own personal computers connected to the Internet [2,4,5]. Elderly males and elderly members of ethnic minority groups lag behind in using the Internet to locate health care information. In 2003, only 11% of African Americans aged 65 and older reported using the Internet for any purpose [2].

### A Cause for Concern

Providing seniors with the requisite skills to use the Internet to locate health information is important for four reasons:

1. Of all medical expenditures in the United States, 40% are for persons 65 and older [6]. With the senior population expected to rise from its current proportion of 12.4% to more than 20% in the year 2030, medical expenditures for seniors will continue to grow.
2. Research by Wenger [7] shows that care for seniors for conditions such as dementia, mobility disorders, pressure ulcers, urinary incontinence, and end-of-life care falls well short of practice guidelines.
3. Americans 65 and older are at constant risk of functional decline by either having to live with a disability or suffering from a chronic illness.
4. The Institute of Medicine [8] and Bach [9] report that substantial disparities exist in the quality of care delivered to ethnic minority patients, who are more susceptible to cardiovascular disease and cancer [10].

The ability to locate relevant health care information benefits seniors by helping them to ask better questions of their health care providers. Several studies show that patients who ask questions, elicit treatment options, express opinions, and state preferences during physician office visits have measurably better health outcomes than those who do not [11-16]. Exposing seniors to Internet-based practice guidelines and standards of care should increase the likelihood that they will receive the proper treatment and take preventive measures.

The question of seniors using the Internet is acutely important in Pittsburgh and the surrounding Allegheny County. Among US counties with populations over 1 million, Allegheny County has the second highest concentration of seniors in the United States, with 17.8% of residents being 65 and older [17,18]. Additionally, research by the University of Pittsburgh Graduate School of Public Health shows that seniors living in Allegheny County have lower levels of computer ownership and Internet access as compared to other demographic groups [19].

The authors hypothesized that teaching seniors to use the Internet to search for health care information and to evaluate the quality of information found would result in (1) reduced reluctance to use computers and increased willingness to use the Internet to find health care information; (2) increased willingness to use external health care information to manage their health care; (3) adoption of a more active role in managing their health care; and (4) increased perception of control over their own health and wellness.

## Methods

This study began in September 2001 with recruitment of volunteers to participate in 5-week training seminars, which lasted through November 2002. One year after completion of the training, participants received follow-up surveys, which concluded in December 2003.

### Training Seminars

One of the authors partnered with Pittsburgh's public library system, a large suburban library, and two senior community centers to sponsor a series of seminars designed to teach seniors to search the Internet for health care information. Holding the sessions in libraries and community centers afforded Internet access to seniors who do not own computers or have Internet access at home. The choice of training centers provided access to a wide range of individuals from varied ethnic groups and socioeconomic status [4]. Participants met for 2 hours, once a week, for 5 weeks. The presenters focused on helping participants use the Internet to learn more about diseases, treatment options, and the health care system, covering the following topics:

- Using a computer and Web browser to access the Internet
- Locating health related information using search engines
- Evaluating health information found on the Internet
- Finding specific types of health information (eg, treatments, medications, physician background and education)
- Using various relevant, high-quality websites (eg, MedlinePlus, ClinicalTrials.gov, OncoLink, IntelliHealth, American Medical Association)

The sessions used constructivist teaching techniques and self-directed learning with a focus on practicing problem-solving skills. Class size was limited to 12 participants to enable instructors to provide more personalized attention.

The overarching goal of the instruction was to encourage seniors to learn more about their health problems, evaluate their health care, and take a more active role in managing it.

### Participant Recruitment

Participants were recruited using posters and flyers targeted to seniors and were available at libraries, senior centers, and other training sites. Ads were placed in senior newsletters and regional publications, and community newspapers. Notices for the sessions were placed in senior center catalogs and program announcements. Also, library and senior center staff members, as well as past participants, were encouraged to spread the word about the program. Any interested senior was allowed to attend the sessions.

### Data Collection, Instruments, and Analysis

As participants began the training sessions, they were asked to complete a baseline questionnaire composed of the Multidimensional Health Locus of Control (MHLC) Scale, the Krantz Health Opinion Survey (HOS), and the Lau, Hartman, and Ware Health Value Survey, as well as the Health Participation Survey. At the end of the 5-week training sessions, participants were asked to complete the same battery of instruments, with the exception of the Health Participation Survey. One year after the training, the 42 participants who completed the training were mailed paper copies of a questionnaire, including the HOS, Lau, Hartman, and Ware Health Value Survey, and Health Participation Survey, as well as 10 additional questions comprising an Internet Use Survey. MHLC was not included in the 1-year follow-up in order to make the questionnaire less daunting to participants. The mailing included a cover letter with instructions and a pre-addressed, postage-paid envelope to return the completed surveys.

### Statistical Analysis

Unless otherwise noted, paired *t* tests were used to compare participants' (completers') baseline scores to 5-week follow-up scores. Where indicated, to account for the missing data from the 18 participants who did not complete the training program, a last observation carried forward imputation was used to analyze all significant results.

### Multidimensional Health Locus of Control (MHLC) Scale

The MHLC Scale [20] was adopted to assess the participants' perception of control over their own health and wellness, or locus of control. The concept “locus of control” was first derived from Rotter's social learning theory, which states that behavior is a function of the expectancy that a specific action will lead to a specific goal or outcome, combined with the reinforcement value of that goal or outcome [21]. Locus of control has three domains: internal, external, and chance. In terms of personal health, an individual with an *external* locus of control believes that the actions of another individual determine her health status. A person with an *internal* locus of control believes her own actions determine her health status. An individual with a *chance* locus of control believes that chance plays a major role in her overall health status.

Previous research found that senior women who used the Internet to locate health information already had an internal health locus of control [4]. However, it was hypothesized that most participants would have an external health locus of control because research shows that older adults generally allow physicians and other health professionals to control their health care [22-28].

### Krantz Health Opinion Survey (HOS)

The HOS [29] was used to measure seniors' desire for more health information, as well as their desire to engage in self-treatment. This survey consists of 16 items yielding scores for health information seeking, behavioral involvement, and an overall score which measures composite attitudes toward treatment approaches. High scores on each subscale represent an individual's desire to be informed on issues regarding their health and a desire to engage in self-care activities. It was hypothesized that participants would initially score low on each subscale as well as the overall score. It was also predicted that scores would increase once participants received instruction on how to use the Internet to locate health information. Furthermore, the authors anticipated that scores would remain stable over the course of a year from the time participants received initial Internet training.

### Health Value Survey

The four-item Lau, Hartman, and Ware Health Value Scale [30] was used to measure the value participants place on their health. Health value is important because, as Wallston and Wallston explain, “There is no theoretical reason to expect health locus of control to predict health behavior, unless it is used in combination with a measure of health value” [31]. Individuals who value their health, whether healthy or suffering from chronic illness, will be more likely to use the Internet to locate and use health information.

### Health Participation Survey

This survey was administered to measure seniors' levels of participation in managing their health care. For example, the first question asked them to rate their level of participation during their last visit with their primary care provider. Question two asked participants to identify the role they played at their last office visit: did they let their health care provider make all the decisions, did they make all the decisions and ask their health care provider to state his/her opinions, or did they take a collaborative role with their provider? Other questions included whether or not they prepared a list of questions for their office visit, how many questions they asked at the last office visit, did they do any research to prepare for their last office visit, and whether they had ever used the Internet to locate health information.

### Internet Use Survey

This survey was administered only at 1-year follow-up. It was designed to measure the impact the Internet had on participants' health care behaviors. Five questions from the Health Participation Survey appeared on this survey but used slightly different wording. Participants were asked to evaluate, on a 5-point scale, their levels of participation with physicians and their use of health information to prepare for physician office visits, change personal behaviors, and make health care decisions.

Ten of the questions were based on a national survey conducted by Baker et al [32]. The questions evaluated the influence Internet-based health information had on participant understanding and decision making regarding a health-related issue. Responses to these questions included a 6-point scale from “Strongly Disagree” to “Strongly Agree.”

## Results

### Participants

A total of 60 participants began the Internet training program, and 42 completed the 5-week training seminar. These 42 participants also completed the baseline and 5-week follow-up MHLC and HOS surveys. Participant makeup consisted of 34 (81%) females and 8 (19%) males. The average age of participants was 72 years, and 34 participants (81%) reported that they were retired. The respondents showed a much higher percentage of computer ownership than typically found in senior populations. Of the 42 participants, 30 (71%) owned a home computer, 25 (60%) reported having used the Internet, and 24 (57%) had used email. Seventeen (40%) respondents reported that they used the Internet to find health care information prior to the study, and 1 (2%) reported using the Internet to join an online support group. Prior to the study, 27 (64%) participants reported having some type of illness, with a subset of 19 (45%) reporting a chronic illness.

Only 27 participants responded to the 1-year follow-up survey, which included the Internet Use Survey. The attrition rate from the 5-week follow-up to the 1-year follow-up was worse for the women than for the men, with 7 of the 8 males responding at 1-year but only 20 of the 34 females.

### Before-After Analysis of Outcomes

#### Krantz Health Opinion Survey (HOS)

HOS health information seeking scores for the 42 participants showed a statistically significant increase from baseline to 5-week follow-up (mean = 28.0 vs 29.6; *P =* .05). Higher scores on the HOS indicate a greater desire for health information and self-treatment. In a sensitivity analysis, to address nonresponse bias due to the 18 participants who did not complete the training or the 5-week follow-up, a last observation carried forward imputation was used, which included all original 60 participants and assumed that the HOS information seeking scores remained at baseline level for the 18 participants who dropped out. This analysis changed the level of significance slightly (*P* = .051). No statistically significant differences were found for the behavioral involvement subscale (*P* = .65).

#### Multidimensional Health Locus of Control (MHLC) Scale

Male (*P* = .02), female (*P* = .05), and overall participants', (*P* = .005) MHLC chance scores showed statistically significant differences between observed baseline and 5-week follow-up results, suggesting that participants' perceptions of the role chance plays in their health declined (Table 1). Other MHLC scores showed movement after participation in the course, but the differences were not statistically significant.

**Table 1:** MHLC mean scores

		**Internal**			**External**			**Chance**		
	**n**	**Baseline(SD)**	**5-Week Follow-Up(SD)**	***P* value**	**Baseline (SD)**	**5-Week Follow-Up (SD)**	***P* value**	**Baseline (SD)**	**5-Week Follow-Up (SD)**	***P* value**
Male	8	22.25 (3.694)	24.00 (2.673)	.33	22.63 (2.669)	21.75 (3.615)	.61	19.00 (2.619)	15.88 (2.997)	.02
Female	34	24.06 (3.931)	24.12 (3.724)	.90	19.15 (5.040)	19.38 (4.599)	.73	16.44 (4.717)	15.29 (4.131)	.05
All	42	23.71 (3.909)	24.10 (3.519)	.44	19.81 (4.855)	19.83 (4.488)	.97	16.93 (4.485)	15.40 (3.914)	.005

In a sensitivity analysis, we included the 18 participants who did not complete the training or 5-week follow-up, assuming unchanged baseline values for the 5-week follow-up of the dropouts. This changed the previously statistically significant MHLC chance findings to insignificant levels for males (*P* = .43), females (*P* = .75), and overall participants (*P* = .53).

#### Health Value Survey

Baseline mean scores from the Health Value Survey were 18.02 and increased only slightly and nonsignificantly during the 5-week follow-up (18.12, *P* = .80). Of the 27 participants who completed the 1-year follow-up, no statistically significant differences were found from baseline to 1-year follow-up (*P* = .22), or from 5-week follow-up to 1-year follow-up (*P* = .10).

#### Health Participation Survey

The Health Participation Survey asked participants to identify the role they played on their last visit to their physician. There were very few changes from baseline to 1-year follow-up (Table 2). Interestingly, none of the participants reported working together with their physicians to make important decisions.

**Table 2:** Health participation survey (n = 27)

	**Baseline**	**1-Year Follow-Up**	**Chi^2^**	***P* value**
	**No. (%)**	**No. (%)**		
**Role played on last visit to physician**				
I let the doctor make all the decisions and I followed them	7 (26)	4 (15)	.021	.89
I made all the decisions and asked the physician to state his/her opinions	0 (0)	1 (4)	-	-
I played a collaborative role with my physician	20 (74)	21 (78)	1.122	.29
Other	0 (0)	1 (4)	-	-
**How do you prepare for physician visits**				
Prepared a list of questions before visit	16 (59)	8 (30)	1.167	.28
Used Internet to locate information prior to visit	3 (11)	6 (22)	2.220	1.1

The Health Participation Survey also asked participants to report how they prepared for physicians' visits. Although fewer respondents in the 1-year follow-up indicated preparing a list of questions prior to their last visit, they did, on average, ask their health care provider more questions than at baseline (mean = 3 vs 4 questions at baseline vs 1 year, data not shown).

#### Internet Use Survey

Administered at 1-year follow-up, the Internet Use Survey asked participants to rate their levels of participation during their last physician office visit. Ratings were based on a 5-point scale from (1) for “No participation” to (5) for “High participation.” Although the median score increased from 3 at baseline to 4 at 1-year follow-up, a Wilcoxon signed rank test used to compare participant responses showed no statistically significant increase in participation (*P* = .38).

Twenty-one of 27 (78%) respondents to the 1-year follow-up survey indicated that they had used the Internet to find health-related information; 11 respondents reported using the Internet for health information at least weekly. Another 10 respondents indicated that their frequency of use was “other,” which provided an open-ended opportunity for further explanation. Responses included as needed, 3 to 4 times per year, 10 times per year, or no additional information.

Ten questions of the Internet Use Survey focused on the impact Internet-based health information had on participants' decision making. The first four questions related to participants' feelings regarding general health information retrieved from the Internet (Table 3). The remaining six questions (Table 4) were aimed only at the 18 participants who said they were suffering from a chronic condition.

**Table 3:** Internet use survey: general health information, 1-year follow-up survey (n = 27, multiple answers possible)

	**Agree or** **Strongly Agree**	
**Question**	**No.**	**%**
1. Did the Information you found on the Internet improve your understanding of the symptoms, conditions, or treatments in which you were interested?	18	67%
2. Did the information you found on the Internet provide you with the ability to manage your health care needs?	5	19%
3. Did the information you found on the Internet challenge you to seek care from another health care provider or health care facility?	9	33%
4. Did the information you found on the Internet challenge you to change the way you eat or exercise?	11	41%

**Table 4:** Internet use survey: patients reporting chronic illnesses, 1-year follow-up survey (n = 18, multiple answers possible)

	**Agree or** **Strongly Agree**	
**Question**	**No.**	**%**
1. Did the information you found on the Internet help you better understand your chronic condition?	13	72%
2. Did the information you found on the Internet help you manage your chronic condition by yourself?	3	17%
3. Did the information you found on the Internet affect the treatments you use for your chronic condition?	6	33%
4. Did the information you found on the Internet help you manage other health problems without visiting a health care provider?	3	17%
5. Did the information you found on the Internet challenge you to seek care from a different physician, health care provider, or health care facility?	3	17%
6. Did the information you found on the Internet challenge you to change the way you eat or exercise?	7	39%

The majority, 18 of 27 (67%) participants, agreed that the information improved their understanding of a health care topic, but most participants also felt that the information did not help them manage their health care needs, challenge them to seek care from another health provider or facility, or challenge them to change their diet or exercise habits. A similar pattern was observed for participants with chronic conditions, with a majority agreeing that the information allowed them to better understand their health problem, but only a minority reporting that the retrieved health information helped them to manage their chronic condition, affected treatments used to control their condition, helped to manage other conditions, or challenged them to change their diet or exercise.

## Discussion

This study explored the impact of training seniors to use the Internet to locate health information. In examining the viability of this endeavor, the authors chose to focus on four research questions (as stated in the Introduction) to explore how Internet usage may or may not affect a group of seniors' decision-making processes.

### Willingness to Use Computers and Internet

The first question to be answered was whether or not participants would experience a reduced reluctance to use computers and an increased willingness to use the Internet to find health care information. Although 30 of the 42 participants already owned a personal computer at the onset of the study, only 17 (40%) reported having used the Internet to locate health information. A year after receiving Internet training, 21 of the 27 respondents (78%, or 50% of the 42 course completers) reported using the Internet, either weekly or as needed, to locate health information. This suggests that older adults are willing to use personal computers to locate health information.

However, since participants for this study were self-selected, it is likely that they had a greater interest in using the Internet prior to the study than the average senior.

A high number of participants (18 of 60, 30%) did not complete the course (n = 18). Reasons for attrition varied. Some examples included family illnesses, difficulty getting to training sites, and frustration in learning to use computers. Several of the participants were not willing to provide reasons for dropping out of the study. Given that the demographic characteristics of the 18 dropouts were similar to the 42 who completed the study, it seems unlikely that the findings would have been substantially different if the 18 participants had completed the 5-week follow-up survey.

### Willingness to Use External Health Information

The second question sought to determine whether there was an increased willingness among participants to use external health information to manage their health care. The HOS score showed a significant (*P* = .05) increase from baseline to 5-week follow-up, indicating a greater desire for health information as well as for self-treatment. However, the majority of participants did not use the Internet or any information source to prepare for health care provider office visits or to review information after office visits. These results suggest that use of the Internet to locate health information did not increase participants' willingness to use the information to manage their health care. Since the number of participants in this study was relatively low, topics for future research include the following: What factors determine a senior's likelihood to prepare for physician office visits? What factors determine whether seniors value finding and using information in support of their health care?

### Active Role in Managing Their Health

A third question focused on whether Internet use allowed participants to adopt a more active role in managing their health. When asked what role they played with their physician during an office visit, 78% of the participants indicated that they played a collaborative role. Yet, as mentioned above, the participants did not use the Internet to prepare for an office visit or to verify information provided by their physicians after an office visit. Furthermore, participants reported that the Internet did not necessarily help them manage either a general health concern or a chronic condition. The results suggest that, if participants were collaborating with their physicians, they were not using information found on the Internet to promote this process. Future research needs to determine whether this observation indicates a problem with the training methodology or suggests other factors are at work, such as seniors' beliefs about how they should interact with physicians. Another possibility is that, although participants indicate that they collaborate with their physicians, they really are not collaborating, whether due to illness or other situational variables [33-44].

### Locus of Control

A final question examined whether Internet use increased participants' perception of control over their health and wellness. The statistically significant reduction in MHLC chance scores from baseline to 5-week follow-up suggests that health care providers or educators can intervene and shift perceptions about seniors' ability to manage their own health care. However, it may also be that those participants who believed that chance plays a major role in their overall health status were more likely to discontinue the course, biasing the analysis of the observed results. Indeed, inclusion of the 18 participants who dropped out (assuming baseline values for the missing follow-up data) eliminated the significant finding.

It is interesting to note that female participants had a higher internal locus of control score than men, starting with the baseline surveys and continuing through 5-week and 1-year follow-ups. The study results suggest that the Internet is one more tool women can use to maintain their internal health locus of control [4]. It also matches past research showing that women take a more active role in their health care, while men are generally more passive [23,25,33,45,46]. Further research is needed to determine why this gender difference exists.

### Limitations

Results from this study seem to suggest that the training sessions are having a positive impact on participants in several ways. However, the rather small sample size limits the power of this study to detect differences. There was a substantial attrition, with only 42 of 60 participants continuing the course over 5 weeks, and only 27 responding to the 1-year follow-up survey. To determine whether the training sessions yield statistically significant positive changes, it will be necessary to increase the total number of participants as well as the response rate after the sessions. Some possible methods to increase response rates include the following:

1. Decreasing the time between the end of the sessions and the follow-up questionnaires
2. Providing incentives for participants to follow up
3. Asking participants to make a long-term commitment to the study

The biggest limitation was the lack of a control group. Participants in this study were self-selected and could potentially have had a greater inclination to engage in information-seeking behaviors as well as preparation for physician office visits.

### Conclusions

The results of this study suggest that the participants experienced an increased willingness to use personal computers to locate health information. However, it did not translate into a willingness to take a more active role in their health care or to use the Internet when making important health care decisions. Further studies will need to specifically address whether use of the Internet to locate health information is a behavior determined by variables such as gender, computer ownership, economic status, and academic background, or whether situational variables, such as health status, type of office visit, and preferences for participation in one's health, play a significant role.

Finally, future studies should examine the qualitative impact of teaching seniors to use the Internet for health care information. Although the instruments used can show how seniors' behaviors and perceptions are changing in aggregate, it would be equally important to attempt to determine how the participants' attitudes toward their health and health care providers change as they gain information-seeking skills. That seniors' health will decline over time is axiomatic. However, the authors believe that increased understanding of their health can lead seniors to have an increased sense of empowerment, self-worth, and dignity. Studies that address these and other issues would be equally worthwhile.

## Abbreviations

HOS: Health Opinion Survey
MHLC: Multidimensional Health Locus of Control
